# Access to healthcare by undocumented Zimbabwean migrants in post-apartheid South Africa

**DOI:** 10.4102/phcfm.v16i1.4126

**Published:** 2024-02-28

**Authors:** Takunda J. Chirau, Joyce Shirinde, Cheryl McCrindle

**Affiliations:** 1 WITS School of Governance, Centre for Learning on Evaluation and Results Anglophone Africa, University of the Witwatersrand, Johannesburg, South Africa; 2 Department of Public Health, Faculty of Health Sciences, University of Pretoria, Pretoria, South Africa

**Keywords:** undocumented migrant, migrant, Zimbabweans, South African healthcare systems, xenophobia, human rights, Tshwane District

## Abstract

**Background:**

Zimbabwean undocumented migrants rely on the South African public health care system for treatment of non-communicable and communicable diseases, surgery and medical emergency services. A gap remains to understand undocumented migrant experiences at a time when accessing public healthcare has been topical in South Africa.

**Aim:**

This article aimed to describe and understand the experiences, challenges and health-seeking alternatives of undocumented Zimbabwean migrants in accessing healthcare services in Nellmapius in Pretoria.

**Setting:**

The study was conducted at Nellmapius in Pretoria.

**Methods:**

A qualitative descriptive research design was used. Structured interviews with 13 undocumented migrants were conducted by applying purposive and snowballing sampling techniques. The data were thematically analysed.

**Results:**

Migrants reported that the attitudes by healthcare officials suggest unwillingness to provide services to undocumented migrants, aggravating their vulnerability and perennial illness. Migrants faced challenges of discrimination, a lack of professional service delivery, a lack of financial capacity to pay for services and a lack of documentation evoking health-seeking alternatives.

**Conclusion:**

Migrants continue to face challenges while accessing subsidised health care. This study confirms that medical xenophobia is generally present in the public health care centres, at least for the sampled undocumented Zimbabwean migrants. The majority of undocumented migrants cannot afford to pay for private healthcare.

**Contribution:**

The findings of this study inform national, provincial and local healthcare facilities to be ethical and provide dignified quality healthcare to undocumented migrants in line with international practices.

## Introduction

South Africa has been marked by a sharp increase in the number of migrants from its neighbours.^1^ In particular, a declining economy, the lack of job opportunities, and breaches of human rights are factors that contribute to undocumented Zimbabwean migrants needing healthcare in South Africa.^2^ An individual who enters or remains in a country with no necessary documents, such as a residence permit or visa, is said to be an undocumented migrant;^3^ if detected, they may be deported. Undocumented migrants tend to have more problems in regard to accessing healthcare services or obtaining residence or work permits.^4^ Undocumented migrants are relying on the South African public healthcare system for treatment of non-communicable and communicable diseases, surgery and medical emergency services.^5^

The influx of migrants into South Africa is met with hostility, distrust and suspicion resulting in episodes of xenophobia.^6^ The hostility against the migrants is replicated in the healthcare system; other authors termed this as ‘medical xenophobia’.^7^ Both the demands of undocumented migrants and the questions of migration, and health, are not sufficiently addressed by the system. The South African Constitution guarantees migrants access to healthcare. The Constitution, Section 27 (1) (a) and (3), does not discriminate against anyone irrespective of nationality.^7^ Migrants have access to basic healthcare including emergency medical treatment, as indicated in Section (3) of the Constitution. The *Refugees Act of 1998*, Section 27 (g), Health 2007 Circular and the *South African National Health Act (No. 61 of 2003)*, Section 4 (3) (b), are additional laws governing access to healthcare for all in South Africa.^8^

Studies conducted in South Africa raised concern over health professionals, for example, nurses ‘indiscriminately practicing medical xenophobia’ as a result of the inconsistency in implementing different pieces of legislation.^4^ The inspiration and justification for this study came from the need to comprehend and analyse how undocumented migrants in post-apartheid South Africa access healthcare.

The aim of this study was to describe and understand the experiences of undocumented Zimbabwean migrants in accessing healthcare services in Nellmapius in Pretoria. The aim of the study was supported by two subsidiary objectives, namely: (1) to identify and describe the challenges faced by undocumented Zimbabwean migrants in accessing healthcare services in Nellmapius in Pretoria and (2) to identify and describe the health-seeking alternatives of undocumented Zimbabwean migrants in accessing healthcare services in Nellmapius in Pretoria.

## Research methods and design

### Study design

This was a qualitative descriptive research study aimed at better understanding and describing the experiences of undocumented Zimbabwean migrants in accessing healthcare services in Nellmapius in Pretoria.

### Setting

The study was conducted in Nellmapius, which is a high-density suburb in the Northeast of Pretoria within the City of Tshwane Metropolitan Municipality in the Gauteng province of South Africa. The population is mainly dominated by black Africans who constitute 95.6%. Of these 50.2% are males and 49.8% are females.^9^ According to the 2011 census, Sepedi is the main language spoken in Nellmapius.^9^ Nellmapius was chosen because there are many Zimbabweans who live in the high-density suburb. The suburb is accessed through Solomon Mahlangu Drive, the Bronkhorstspruit Road (R104) and Stormvoel Road. It is serviced by different amenities, for example, schools, malls and industries. The industries servicing the area are in Waltloo, for example, Ford Motor Company that provides employment to people in both Nellmapius and Mamelodi. Other suburbs surrounding Nellmapius are Mamelodi, and Savanna Country Estate.

### Study population, sampling strategy and sample size

The target population was migrants from Zimbabwe. Purposive and snowball non-random sampling techniques, which are a type of non-probability sampling, were used to select undocumented migrants to participate in this study. Using purposeful sampling, the researchers were able to gather valuable information about the topic under study from undocumented migrants. Furthermore, snowball sampling technique was used to identify other undocumented migrants – because these are a ‘hidden’ population and not easy to access. The participants met the eligibility criteria of being a Zimbabwean national who has not acquired a visa or asylum or refugee permit provided by the Department of Home Affairs, resides in Nellmapius for less than 15 years, aged over 18 and having used a hospital, community health centre, or public clinic for medical care and were willing to participate. The sample comprised of 13 participants and was determined by data saturation. All the 13 interviews were transcribed.

### Data collection

Interviews lasting approximately 35 min were conducted. The interviews were conducted in English in August 2022 at Nellmapius by the researcher. These interviews were audio recorded, while for others manual note taking was employed as undocumented migrants refused to be tap recorded. A semi-structured interview guide with 18 questions was used to interview migrants about their experiences of using the public healthcare system in South Africa. The guide explored issues of demographic profiles, migration to South Africa, healthcare experiences at facilities, challenges encountered when receiving healthcare services, and health-seeking alternatives. All the interviews were transcribed and exported to ATLAS.ti™ Version 22.2, a programme used for analysing qualitative data.

### Data analysis

Thematic analysis was employed in data analysis and interpretation. ATLAS.ti™ Version 22.2 software was used to manage and store the transcribed information from interviews. Firstly, the researcher started by familiarising himself with the transcribed collected data. Secondly, the researcher started coding of the 13 transcriptions – this included open coding to draw insights from the data. A code list was established and where necessary new codes were developed. The coding process produced 148 codes. Upon completion of coding, the outputs were thematised. This study had no prior hypothesis (deductive); the establishment of codes and themes emerged from the data (inductive). The coding was useful to systematically compare experiences across all participants. Thirdly, the researcher shared the codes and categories with the supervisors, and agreed to develop the analytical framework. Fourthly, the researcher started interpreting the data based on the developed codes and categories of interest and responding to the aim and objectives of the study. The software was effective for the management, storage, coding and retrieval of information.^9^

### Ensuring trustworthiness

To ensure credibility of the study, the researcher depended on the data that were given by those who took part if they represented the right people by confirming their nationality, migrant status, place of stay, number of years residing in Nellmapius, their age and if they have visited a hospital, community health centre, or public clinic for medical care. The researcher gave full details of the study’s purpose and went through the informed consent form for the participant to agree to be part of the study. Furthermore, the researcher and the supervisors went through a debriefing of the data collection process to ensure credibility of the findings. This process, additionally helped to reduce potential biases that the researcher might have brought into data analysis. The researcher also continuously reflected on his positionality throughout the research process.

### Ethical considerations

Ethical clearance to conduct this study was obtained from the University of Pretoria, Faculty of Health Sciences Research Ethics Committee (No. 130/2022). In upholding research ethics, confidentiality of information and anonymity were preserved; quotations in this study make use of a unique code given to each participant.

## Results

The undocumented migrants in this study were of different ages as shown in Table 1. Just over half were between ages of 26 and 35 years. There were nine females and four males. In relation to education, eight completed secondary school, four completed high school and one completed primary school. The emerging themes and sub-themes are presented in Table 2.

**TABLE 1:** Socio-economic demographic data.

Demographic profile	Frequency (*N* = 13)
**Age (years)**	
18–25	1
26–35	7
36–45	4
46–55	1
**Sex**	
Male	4
Female	9
**Education**	
Primary	1
Secondary	8
High	4
**Employment sector**	
Formal	1
Unemployed	11
Informal	1
**Duration of stay in South Africa (Nellmapius)**	
1–5 years	6
6–10 years	6
11–15 years	1

**TABLE 2:** Themes and sub-themes.

Theme	Sub-themes
1. Healthcare experiences	Services received at health facilities
2. Challenges encountered when receiving health care services	Discrimination
	A lack of professional service
	Financial limitation
	A lack of documentation
3. Medical health alternatives and strategies	Spiritual consultations
	Self-medication

### Theme 1: Healthcare experiences

There seems to be agreement among the participants that as long as one is identified as a migrant the treatment received from the health officers is different compared to the people of South African origin. This is despite the kind of services one would want to receive at the health facility. This theme therefore identifies the types of services sought by undocumented migrants at health facilities.

#### Sub-theme 1: Services received at health facilities

**FIGURE 1:**
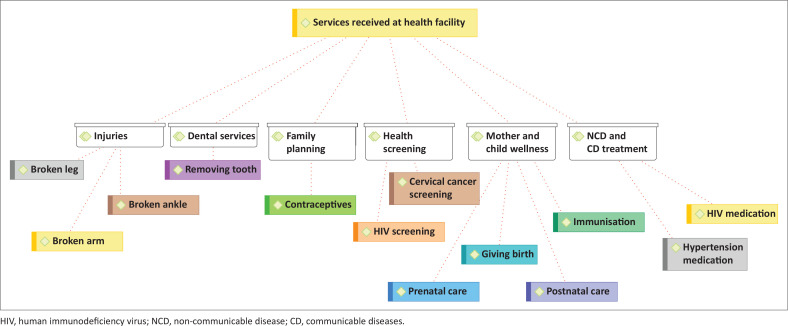
Services at health facilities consulted.

Under this theme, six main codes came out of the qualitative analysis of the interviews to illustrate the services sought by undocumented migrants. The codes were dental services, injuries, family planning, mother-and-child wellness and treatment of non-communicable and communicable diseases. These codes have sub-codes as depicted in Figure 1. Migrants reported that experiences were largely determined by the type of service required. Figure 1 shows ATLAS.ti™ graphic illustration of services at healthcare facilities, which the interviewed migrants received.

On one hand, there are those who indicated to have bad experience. Nurses showed negative attitude towards migrants without any care and compassion while providing medical healthcare. One female participant expressed her frustration concerning her experience at the clinic in Nellmapius; she said:

‘I am visiting the clinic every month to get my treatment, at times I feel that the nurses don’t want to help because they shout at us and blame us for shortage of pills. Because many people are taking the pills at the clinic.’ (UNDZIM 4)

Over eight migrants unanimously agreed that the process of receiving medical care co-existed with disrespect, insults and victimisation from nurses simply because they are not South Africans.

On the other hand, about two migrants reported that they had received quality care and did not succumb to verbal abuse by the nursing staff at the same facilities. However, migrants as a whole, frequently highlighted that their experiences are heavily dependent on the nursing staff attitudes and behaviours on a particular day. This naturally led to good or bad experience.

Migrants (4 out of 13) from time to time were asked to pay for services at Mamelodi Regional hospital. This is despite free subsidised public healthcare in South Africa. One female participant said:

‘On 25 February 2022 went to Daga/Mamelodi hospital and I was asked to pay money for operation. I was asked to pay R12 000.00 for that operation and also the number of days I spent in the hospital. For me to be discharged my husband had to pay R1200.00. When I went back for check-up, I was again asked to pay for R800.00 to be treated …’ (UNDZIM 7)

These migrants (11 out of 13) are unemployed and struggle to sustain their individual and household needs and yet are expected to pay for their healthcare. There are those who were outrightly denied treatment because of the lack of a visa or permit. The denial of quality care is a threat to public health, because some suffer from communicable diseases that easily spread within the community:

‘The horror inside the maternity ward.’

Female migrants were disheartened by the way they were treated inside the maternity ward at Daga hospital. Female migrants reported that they were harassed and emotionally tortured by the actions, attitudes and behaviours of the nurses. One female migrant stated:

‘When I was pregnant, I was transferred from Extension 1 clinic to Daga hospital to give birth. The service was not the same with South Africans … There were no check-ups from the nurses. But for others in the ward, they were checked from time to time. The sister only came when I was giving birth … I only started getting attention when the baby was coming. I was not happy with the way I was treated at the hospital. They ignored my pains but I had to be strong because I feared that they can be rude to me.’ (UNDZIM 3)

The majority of the participants (8 out of 13) reported that they were given less priority compared to South Africans. As a result of this stigmatisation and discrimination, the interviewed migrants repeatedly indicated that they had given up going to seek medical care. This contributed to more vulnerability among migrants particularly among females compared to males. This is because females visit health facilities frequently.

### Theme 2: Challenges encountered while receiving healthcare services

Four codes came from the qualitative analysis of the interviews to showcase the barriers that migrants experience while seeking healthcare. The codes are discrimination, a lack of professional service, financial limitations and a lack of documentation.

#### Sub-theme 1: Discrimination

Many migrants shared personal stories about being discriminated against. The majority of migrants reported that healthcare workers prioritised South African people compared to foreign nationals. Two sub-themes contributing to that were cited as the migrant status (undocumented) and nationality (country of origin). One immigrant said the following:

‘… Not at all, the treatment of the locals is not the same as the foreigners. If you speak English they will ask where you come from and if you are not from South Africa their attitude change and you can see that they don’t want to help you.’ (UNDZIM 7)

#### Sub-theme 2: A lack of professional service

Under this theme of professionalism, four other sub-themes emerged from the analysis, namely language barrier, attitude of nurses, long queues, and harassment. This study only discusses three critical ones next.

Nurses spoke in their vernacular language, for example, isiZulu and seTswana, which the majority of the migrants neither speak nor hear. The majority of these migrants are fluent speakers of English. Migrants reported that the impact of this is misinterpreting and misunderstanding instructions in particular those who have to do with medication dosage.

The migrants spoke openly and were conscious of the negative attitudes and behaviours of the nursing staff. Migrants believed that the attitudes and behaviours of nurses inculcated fear and frustration whenever they visited the health facilities. Migrants, collectively agreed that their own and that of fellow migrants’ negative experiences with the nurses were both a deterrent and a motivator to seek alternative healthcare. They, therefore, reported that they would consider carefully if any family member strongly needed medical attention.

Migrants reported that they encountered excessive waiting periods as a result of long queues. Overall, they described that the attitude and behaviours of the nurses and other health staff, for example, receptionists are linked to inefficiency. The migrants also reported that long waiting hours at health facilities had implications for their livelihood strategies. This is because waiting time costs them hours of productive work, causing them to lose money. To explicate on this, one frustrated female participant highlighted that:

‘Aaa! Extension 1 clinic, people get up at 4 am to go to the clinic. The queue is usually very long and you stay the whole day without being attended to. If you are not attended to on that day, they ask you to come the following day, and you have to wake up every early. Two days can go by without being attended.’ (UNDZIM 7)

#### Sub-theme 3: Financial limitation

Eleven out of 13 interviewed participant migrants reported that the form of employment available for migrants is casual labour. This work is *ad hoc*, insecure and less remunerative compared to formal employment that requires permits. This study sees this as nothing less than a migrant population trapped in a ‘vicious cycle of poverty’. The failure to regularise their stay plays a significant role in reinforcing this. From time to time migrants reported they were asked to pay for health services in particular, the emergency cases. The migrants claimed that they were unable to pay the clinic and hospital out-of-pocket fees. During the interviews, they often frequently reported that they were poor.

#### Sub-theme 4: A lack of documentation

Four migrants believed that the lack of documentation particularly visa or permit and passport contributed to the myriad challenges they faced. They reported that healthcare workers now and again asked for valid permit to access healthcare. The failure to produce that led to the denial of treatment or being requested to make a payment. Migrants described that having a passport is key to identification and opening a clinic and/or hospital file for medical records. Table 3 shows the number of times a challenge to accessing care was coded in the interviews.

**TABLE 3:** A summary of challenges to accessing medical care.

Code group	Codes	Frequency (*N* = 13)
Discrimination	Migrant status (undocumented)	3
	Xenophobia	3
	Nationality (country of origin)	2
A lack of professionalism	Language barrier	10
	Attitude of nurses	23
	Long queues	1
Financial limitation	Out-of-pocket payment	2
A lack of documentation	No passport	2
	No permit or visa	2

### Theme 3: Medical health alternatives and coping strategies

**FIGURE 2:**
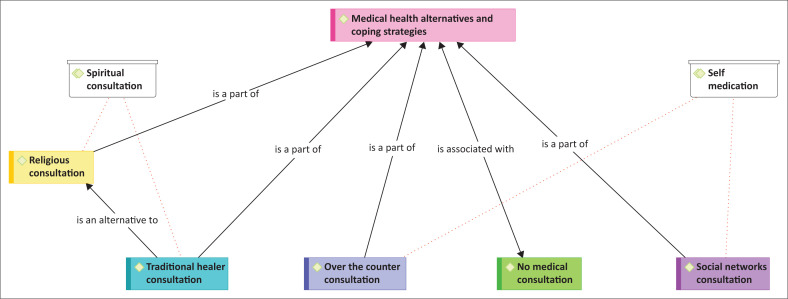
Health alternatives and coping strategies.

Through the qualitative analysis of the interviews, two important sub-themes evolved to describe the migrants’ medical health alternatives and coping strategies, namely spiritual consultations (with sub-theme of religious and traditional consultations), and self-medication (with sub-themes of social network consultation, over-the-counter consultation and not seeking medical attention). Critical to the aforementioned is that migrants are not submissive victims of their circumstances – the strategies foreground their agency and ingenuity. Figure 2 is an ATLAS.ti™ graphic illustration of the two themes.

#### Sub-theme 1: Spiritual consultations

Migrants reported that they relied on prayer led by their church leaders. The most commonly cited church was the African Apostolic Indigenous Church – such churches are prevalent in Nellmapius. In the local Zimbabwean Shona vernacular, the members are referred to as *mapostori*, meaning apostles. Migrants reported that they believed in the power of holy spirit to restore good health and well-being. The migrants reported to have received prayers, holy water, stones and other remedies to get healing.

Apart from spiritual consultations, migrants reported that they visited traditional healers (locally known as *sangoma*). The migrants described that at *sangoma* they received herbs, roots and other traditional medications. For about five migrants, a *sangoma* served as both the first and last line of defence against the crippling illnesses migrants encountered. Furthermore, migrants expressed that spiritual consultations did not require cash up front; however, they often gave a token of appreciation – a small fee.

#### Sub-theme 2: Self-medication

Migrants said that from time to time they visited the pharmacy to get over-the-counter medication. They reported that upon visiting a pharmacy, they explained their symptoms to the pharmacist. However, migrants were aware of the dangers of self-diagnosis and treatment that it could worsen their illness. All the 13 migrant participants reported that they had no medical insurance and had to pay for their medication; this is despite inadequate income.

Migrants expressed that social networks remain networks of necessity. The migrants further explained that they relied on one another to provide unfinished medications – where symptoms were the same thereby saving money and also preserving their dignity that could be violated at health facility. To say that social networks are essential is to emphasise their value as a means of support for a living. Migrants expressed that they do not network and interact just out of necessity, but in reality, value the spirit of *ubuntu* while sojourning.

Participants described that their own or fellow migrants’ unpleasant healthcare experiences influenced or contributed to not seeking healthcare. Over half of the participant migrants indicated that it was paramount to preserve their dignity and not be harassed while seeking treatment as the humiliation leads to frustration and stress.

## Discussion

This study explored experiences, challenges and health seeking alternatives of undocumented Zimbabwean migrants in accessing healthcare services in Nellmapius in Pretoria. Migrants’ experiences regarding access to healthcare and challenges of discrimination, a lack of professional service, financial limitation and a lack of documentation as well as coping strategies of spiritual and self-medications formed the core themes. These identified themes build and complement experiences, challenges and health seeking alternatives in previous studies.^7,10^

The theme of healthcare experiences and services received by undocumented migrants expands on other studies that found that undocumented migrants are not accessing quality care compared to South Africans.^5^ This analysis, therefore, elucidates that primary health care is the most sought service at healthcare facilities by the migrant participants. Literature has described the harrowing experiences of migrants trying to receive different levels of care in South Africa.^7,10,11^ Despite the right to healthcare guaranteed by the South African Constitution, the *National Health Act*, and Department of Health circulars, this study uncovered that they have been denied because of policy and practice incoherence and discrimination. These findings support other studies indicating that migrants have succumbed to medical xenophobia, which is institutionalised and embedded in the healthcare system^5,12^ – a contravention of their constitutional right. Institutionalised challenges indicate inefficiency of the health system. Challenges such as long waiting periods do affect both South Africans and undocumented migrant patients.^5^

The lack of uniform application of the law indicates a lack of systematisation with regard to undocumented migrants’ access to healthcare. According to the analysis of the interviews, it is difficult for undocumented migrants to obtain healthcare because the sources of their difficulties lie at distinctive levels of the system – indicating that complex issues require complex solutions. Nurses are forced to act according to their personal convictions because there are no guidelines for treating illegal migrants.^13^

This study affirms that migrants were not immune to infectious diseases, for example, human immunodeficiency virus (HIV) and acquired immunodeficiency syndrome (AIDs), sexually transmitted infections and tuberculosis.^7^ If these are untreated, there is likely to be an epidemic in communities where they reside. Previous studies have shown that migrants adopt strategies to avert challenges, for example, adoption of healthy behaviours, adaptation, delaying treatment and self-treatment.^11,14^ This has negative health outcomes. Such findings are corroborated by this study. The uncontrolled in-take of medication can lead to disease resistance,^15^ and death. This study may inform public health interventions aimed at improving access to quality and dignified healthcare to migrants.

Despite the study’s contributions, some limitations must be acknowledged to provide various prospective possibilities for further exploration. Conducting interviews with nurses would have given insight into policy and practice relating to undocumented migrants. Besides that, a balanced analysis could have been derived by interviewing nurses to understand their justification to the said ‘medical xenophobia’. Snowballing of migrants may be a possible source of bias if migrants choose other migrants with same experiences resulting in unanimity of testimonies.

Being a qualitative research, there was no absolute claim that the experiences of undocumented migrants in Nellmapius are comparable to those outside of it. Neither does this study suggest that undocumented Zimbabwean migrants are the only migrants who are denied access to subsidised public healthcare in South Africa. The study could have purposively sampled other undocumented migrants from Malawi, Mozambique and the Democratic Republic of the Congo to study their experiences, challenges and draw conclusions. Additionally, this could have enabled generalisations about undocumented migrants’ access to subsidised public healthcare in South Africa. Using the tape recorder while conducting fieldwork presented a significant obstacle. During the interview process, some rich data may have been lost in manual note taking, transcribing and trying to listen simultaneously.

This study does not wish to classify all the health officials as abusive and mistreating migrant patients from Zimbabwe. Even if the generalisations presented here are accurate at the scale of abstraction at which they are set, they ought to be supplemented by an acknowledgement that somehow there exist variances. Migrants’ own, and other fellow Zimbabwean experiences may have an influence on negative health seeking alternatives, which could perpetuate illnesses – particularly as a result of the lack of treatment of communicable diseases.

This study demonstrates the significance of standard operating procedures that agree with legal frameworks that can enable undocumented migrants to receive healthcare and provide guidance to healthcare providers on how to give quality and dignified care to migrant patients. Overall, the findings of this study, and other studies, remain somewhat the same; no unified approach has been developed by the government to deal with the issue. This is indicative of inefficiencies to resolve the impasse around access to health by undocumented migrants. Systemic and ongoing barriers to receiving public healthcare indicate that the South African administration has failed to meaningfully address the problem. Consequently, this study is a situational analysis that evaluates the relevant applicability and implementation of South African immigration policy, department of health circulars, and national health insurance policy in relation to the availability of primary health care for undocumented migrants in South Africa, Zimbabwean migrants being no exception.

## Conclusion

This study sheds light on the experiences of undocumented migrants in Nellmapius in Pretoria who depend on the subsidised public healthcare facilities for treatment. The results provided here are intended to better understand the public healthcare issues experienced by undocumented migrants from Zimbabwe. The findings add to a small but increasing body of knowledge on undocumented migrants’ healthcare access. By investigating the access to healthcare of 13 undocumented Zimbabwean migrants, this study has shown that their experiences are not homogeneous, and they face a myriad of challenges that are rooted in the system itself, discrimination, attitudes, and behaviours of nurses inducing alternative health seeking behaviours.
