# Corrigendum: Access to healthcare by undocumented Zimbabwean migrants in post-apartheid South Africa

**DOI:** 10.4102/phcfm.v16i1.4625

**Published:** 2024-05-31

**Authors:** Takunda J. Chirau, Joyce Shirinde, Cheryl McCrindle

**Affiliations:** 1 Centre for Learning on Evaluation and Results Anglophone Africa, Faculty of Commerce, Law and Management, University of the Witwatersrand, Johannesburg, South Africa; 2 School of Health Systems and Public Health, Faculty of Health Sciences, University of Pretoria, Pretoria, South Africa

In the published article, ‘Chirau TJ, Shirinde J, McCrindle C. Corrigendum: Access to healthcare by undocumented Zimbabwean migrants in post-apartheid South Africa. Afr J Prim Health Care Fam Med. 2024;16(1):a4126. https://doi.org/10.4102/phcfm.v16i1.4126’, there was an error in affiliations for all 3 authors.

Instead of WITS School of Governance, Centre for Learning on Evaluation and Results Anglophone Africa, University of the Witwatersrand, Johannesburg, the first author, Takunda J. Chirau’s affiliation should be, Centre for Learning on Evaluation and Results Anglophone Africa, Faculty of Commerce, Law and Management, University of the Witwatersrand, Johannesburg, South Africa.

Instead of Department of Public Health, Faculty of Health Sciences, University of Pretoria, Pretoria, the co-authors, Joyce Shirinde and Cheryl McCrindle’s affiliations should be, School of Health Systems and Public Health, Faculty of Health Sciences, University of Pretoria, Pretoria, South Africa.

The authors apologise for this error. The correction does not change the significance of study’s findings or overall interpretation of its results or the scientific conclusions of the article in any way.

